# Ahmed’s sub-flap mattress suture deep sclerectomy assessment with Ultrasound Biomicroscopy

**DOI:** 10.1007/s00417-024-06598-4

**Published:** 2024-10-17

**Authors:** Mina Maged Habib, Gihan Mohamed Hilmy, Ahmed Mostafa AbdelRahman, Mohamed Sabry Kotb

**Affiliations:** Ophthalmology department, Kasr Alainy, Faculty of Medicine, Cairo University, 3 Montaser Housing, Elaharam, Cairo, Giza Egypt

**Keywords:** Deep sclerectomy, Ahmed’s sub-flap mattress suture, UBM, IOP

## Abstract

**Purpose:**

To assess the efficacy of adding Ahmed’s sub-flap mattress suture to deep sclerectomy (DS).

**Methods:**

Forty eyes with open angle glaucoma were assigned randomly into two groups: *Group A*: underwent DS with Ahmed’s sub-flap mattress suture. *Group B*: underwent conventional DS. Patients were followed up closely for 6 months with serial IOP measurements and ultrasound biomicroscopy (UBM) was used to assess the surgical site functionally and anatomically at the first and sixth month.

**Results:**

Adding Ahmed’s sub-flap mattress suture improved the IOP lowering effect of DS significantly from 43% in group B to 53% in group A at 6-month (*p* = 0.027). IOP in group A was at 1 week, 1 month and 6-month visits (7.9 ± 1.3, 11.7 ± 2.2 and 13.3 ± 1.9 mmHg respectively) compared to group B (10.1 ± 4.6, 14.1 ± 5.2 and 16.8 ± 4.1 mmHg respectively) (*p* = 0.025, 0.041 and 0.001 respectively). UBM parameters were significantly larger in group A at 1 and 6 months. Strong statistically significant negative correlations were established between IOP and all the UBM parameters apart from intrascleral lake height at the first and sixth month (*p* < 0.01 in all of them). Finally, significant correlations were found between IOP at 6 months and whole bleb anteroposterior length and height at 1 month (*p* = 0.001).

**Conclusion:**

Adding Ahmed’s sub-flap mattress suture to routine DS is an effective economical addition that will enhance the IOP lowering effect of DS. Also, assessment of the bleb by UBM is useful in predicting the success of deep sclerectomy surgery.

## Key messages

***What is known:***

- Ahmed’s sub-flap mattress suture proved to be an effective economical addition to deep sclerectomy.

***What is new***:

- UMB parameters are significantly larger in Ahmed’s sub-flap mattress suture deep sclerectomy than in conventional deep sclerectomy.
- UBM parameters in early postoperative period in deep sclerectomy can be used as indication for late success or failure.

## Introduction

Primary open angle glaucoma (POAG) can be treated either medically or surgically. While medical treatment is usually of higher safety profile to the patients, its efficacy is usually hindered by compliance issues. On the other hand, surgical intervention provides better long-term control of intra-ocular pressure (IOP), despite its surgical possible complications [1, 2].

Sub-scleral trabeculectomy (SST) was considered the mainstay of surgical treatment of POAG since 1968 [3], but it has many intra-operative and post-operative complications like: hypotony and higher risk of infection. So, more surgeons now are adopting the use of non-penetrating glaucoma surgeries that have a higher safety profile in POAG, such as: non-penetrating deep sclerectomy (NPDS), visco-canalostomy and canaloplasty [4, 5].

Many modifications were made to NPDS to enhance its IOP lowering effect by adding absorbable and non-absorbable spacers while still maintaining the superior safety profile. Most of these options are costly and not widely available in our practice in Egypt as a developing country [6].

In this study, we investigated the efficacy of adding Ahmed’s sub-flap mattress suture [7] to routine NPDS by comparing the IOP lowering effect and using the ultrasound biomicroscopy (UBM) to assess the bleb and the intrascleral lake at the surgical site and to study the correlation between the UBM parameters with IOP.

## Methods

This is a prospective randomized controlled trial to assess the efficacy of adding Ahmed’s sub-flap mattress suture to NPDS and to compare it to the conventional NPDS using UBM. The study was conducted in the Ophthalmology Department in Cairo university hospitals.

A total of 40 eyes of 28 patients with open angle glaucoma not controlled on medical treatment, were recruited from Kasr-Alainy glaucoma subspecialty clinic at Cairo university hospitals. All patients signed an informed consent for the surgery and participation in the study after they received a detailed explanation of the benefits expected and the possible risks of undergoing the surgery. The study was performed according to the tenets of the Declaration of Helsinki and was approved by the ethics Committee of the Faculty of Medicine, Cairo University.

Patients were randomized into two groups, each containing 20 eyes using Excel-created random numbers, group A who underwent deep sclerectomy with Ahmed’s sub flap mattress suture and group B who underwent standard deep sclerectomy.

We excluded patients with a history of glaucoma surgery, laser trabeculoplasty or refractive corneal procedures. Also, patients presenting with angle closure glaucoma, uveitic glaucoma, aphakic glaucoma, silicon induced glaucoma and neovascular glaucoma were excluded.

Full history was taken including history of systemic or ocular diseases, ocular surgeries, number and duration of anti-glaucoma medications used. Then, complete ophthalmological examination was conducted including visual acuity assessment, slit-lamp anterior segment examination, IOP measurement using Goldmann applanation tonometer GAT (HAAG-STREIT AT 900, Edinburgh Way, Harlow, UK), fundus examination using slit lamp biomicroscopy and 90-D lens (Volk, VOLK Optical; Mentor, Ohio, USA) and finally gonioscopy using three-mirror Goldmann contact lens (Volk, VOLK Optical; Mentor, Ohio, USA) to confirm open angle. Perimetry and optical coherence tomography of optic nerve head (OCT ONH) were performed to confirm the anatomical and functional damage by glaucoma.

All the surgeries were performed by 3 surgeons (M. H., M. K. and A. A.) under peri-bulbar anesthesia. Each surgeon did an equal number of each group to avoid bias. In group B (standard deep sclerectomy, 7/0 Vicryl corneal traction suture was applied to the superior cornea to provide better exposure to the surgical site followed by limited periotomy using Vannas scissors to create a 10 mm fornix based conjunctival flap. Limited diathermy was applied for hemostasis. Dissection of a 4 × 4 mm partial thickness scleral flap (nearly 50% of the scleral thickness) hinged at the limbus extending 1.5 mm into the clear cornea was done using a crescent knife. Sponges soaked with Mitomycin-C (MMC) ^®^ with a concentration of 0.4 mg/ml were applied to the sub-flap and the subconjunctival space for 2 min then the whole area was thoroughly washed with balanced salt saline solution. Using a microvitreoretinal blade (MVR), paracentesis was created then 0.2 mm of intracameral 1% acetylcholine was injected to constrict the pupil. The deep scleral flap was dissected starting just inside the edges of the superficial flap using a crescent knife and the dissection was carefully continued centrally to deroof the Schlemm’s canal and expose the trabeculo-Descemet’s membrane (TDM). Peeling the inner wall of the Schlemm’s canal using a blunt tipped forceps was sometimes needed to enhance the aqueous percolation. Careful excision of the deep flap at its base was done using Vannas scissors. Repositioning of the superficial flap was done with two 10/0 Nylon sutures at corners. The conjunctiva was reapproximated using two watertight 10/0 Nylon sutures. Finally, combined antibiotic and steroid eye ointment is applied to the ocular surface followed by sterile patching of the eye.

**Fig. 1:**
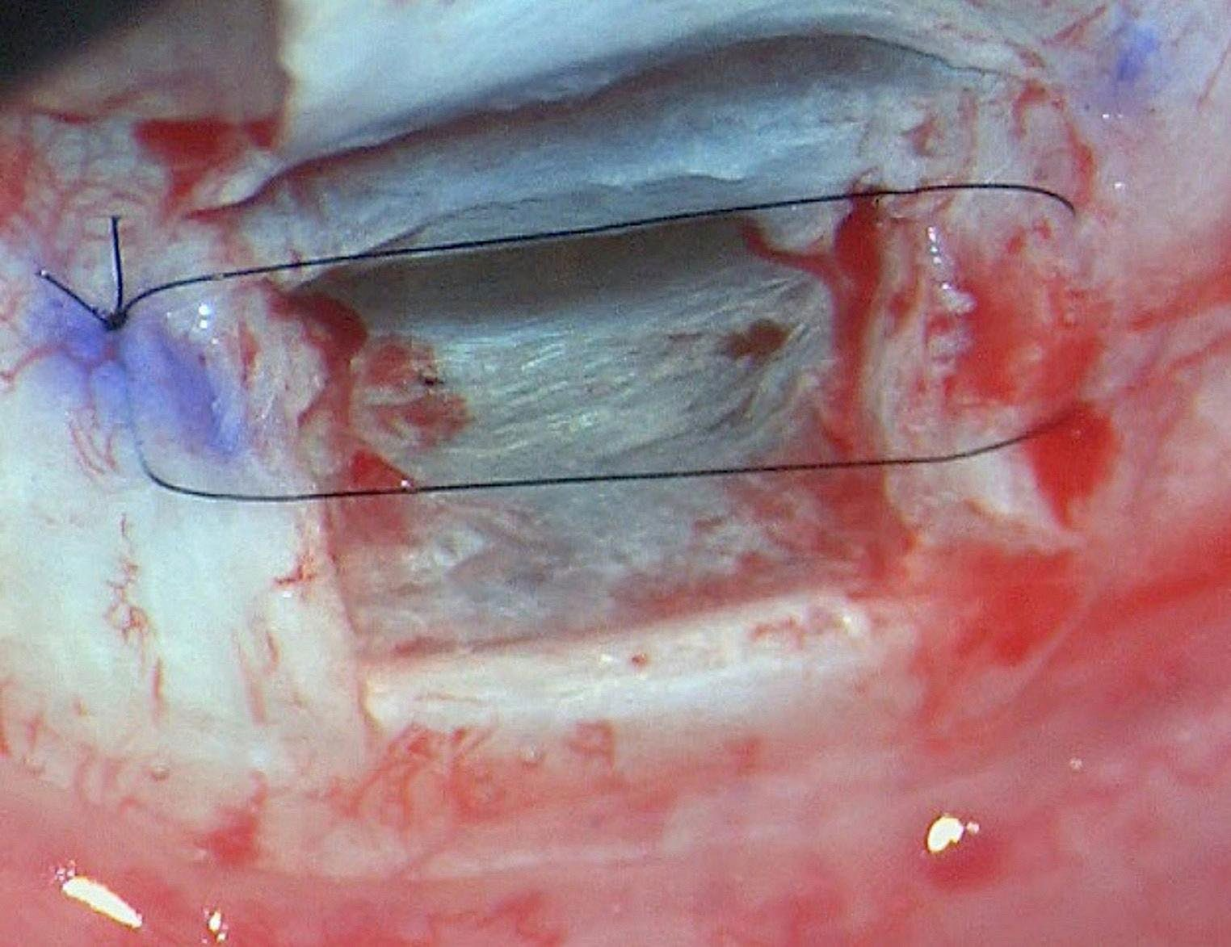
Ahmed’s sub-flap mattress suture in place with its proximal limb over the floor of canal of Schlemm

In group A (deep sclerectomy with Ahmed’s suture), same steps of the standard DS were done till the excision of the deep flap is done. Then, one 10/0 Nylon (Alcon)^®^ mattress suture was applied beneath the superficial flap with its proximal limb passing over the floor of the canal of Schlemm then the suture is embedded using McPherson forceps (Fig. 1).

Post-operative treatment for both groups was topical Prednisolone Acetate ophthalmic suspension and Gatifloxacin antibiotic drops. Drops were applied 5 times daily and tapered gradually over 1 month.

All patients were followed up for 6 months with a minimum of 4 post-operative follow-up visits after 1 week, 1 month, 3 months and 6 months. In each visit, IOP was measured, the bleb was assessed, the anterior chamber and the fundus were examined.

Complete success was defined as an IOP of 6 to 18 mmHg without post-operative anti-glaucoma topical medications, and a qualified success as the same IOP range with post-operative topical medications.

**Fig. 2:**
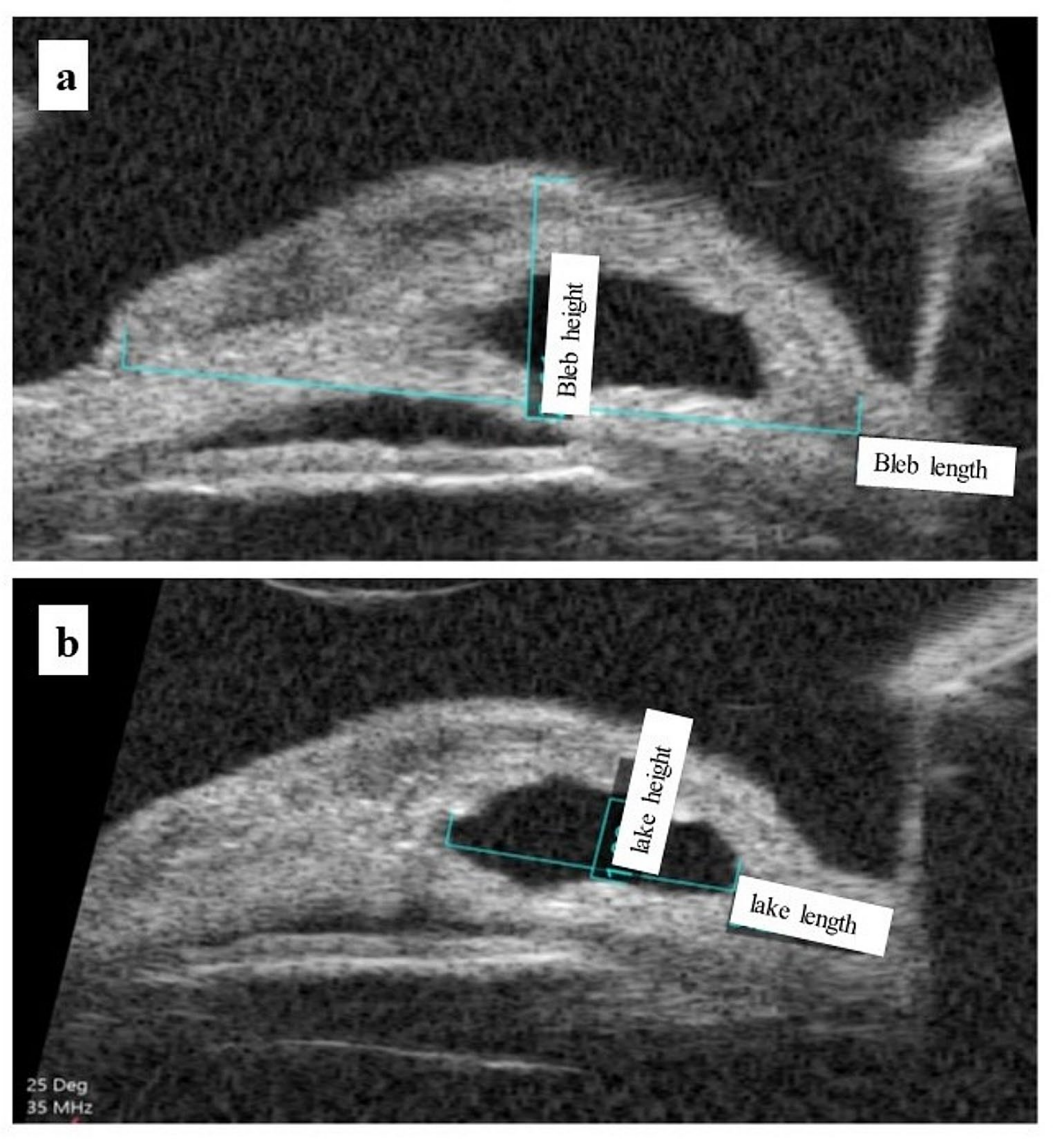
Dimensions measured by UBM: **a** -bleb length: whole anteroposterior length of the conjunctival bleb. -bleb height: the height of the conjunctival bleb. **b** -lake length: anteroposterior length of the intrascleral lake. - lake height: the height of intrascleral lake

UBM was done at 1 month and 6 months by the same operator (G.M.H). For each UBM scan, the following parameters were measured: The whole bleb’s maximum anteroposterior length and height (Fig. 2A) and the intrascleral lake’s maximum height and length (Fig. 2B).

## Results

Regarding demographics, no statistically significant differences were found between the two groups regarding age, gender, pre-operative IOP, pre-operative antiglaucoma medications, cup to disc ratio by fundus examination, corrected visual acuity and angle grading by gonioscopy (Table 1).

**Table 1:** Demographic and pre-operative comparative analysis

	Group A				Group B				*p* value
	Mean	SD	Min	Max	Mean	SD	Min	Max	
Age in years	44.3	16.4	18	75	44.6	11.9	18	62	0.946
Gender (M/F)	15/5				11/9				0.185
Pre-op IOP in mmHg	30.3	8.4	22	46	30.1	4.5	21	40	0.786
Pre-op meds	3.2	1.2	1	5	2.82	1.1	1	5	0.280
C/D ratio	0.83	0.10	0.6	0.95	0.82	0.09	0.7	0.95	0.770
CVA	0.56	0.28	0.05	1	0.51	0.24	0.05	1	0.713
Angle	3.7	0.47	3	4	3.6	0.50	3	4	0.513

**Fig. 3:**
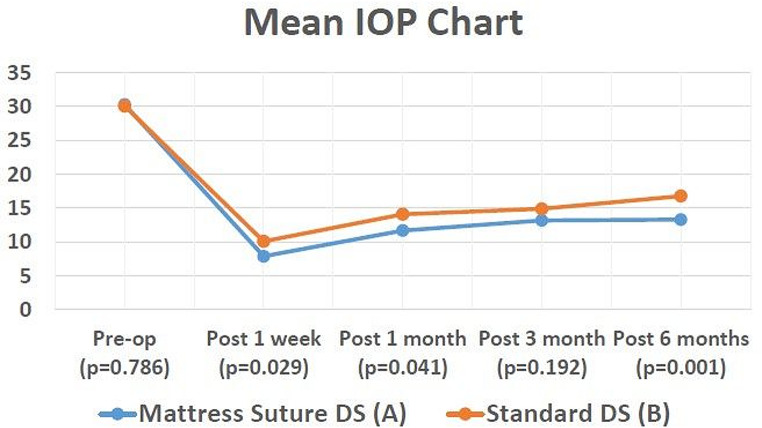
IOP changes throughout the follow up period in both groups

**Fig. 4:**
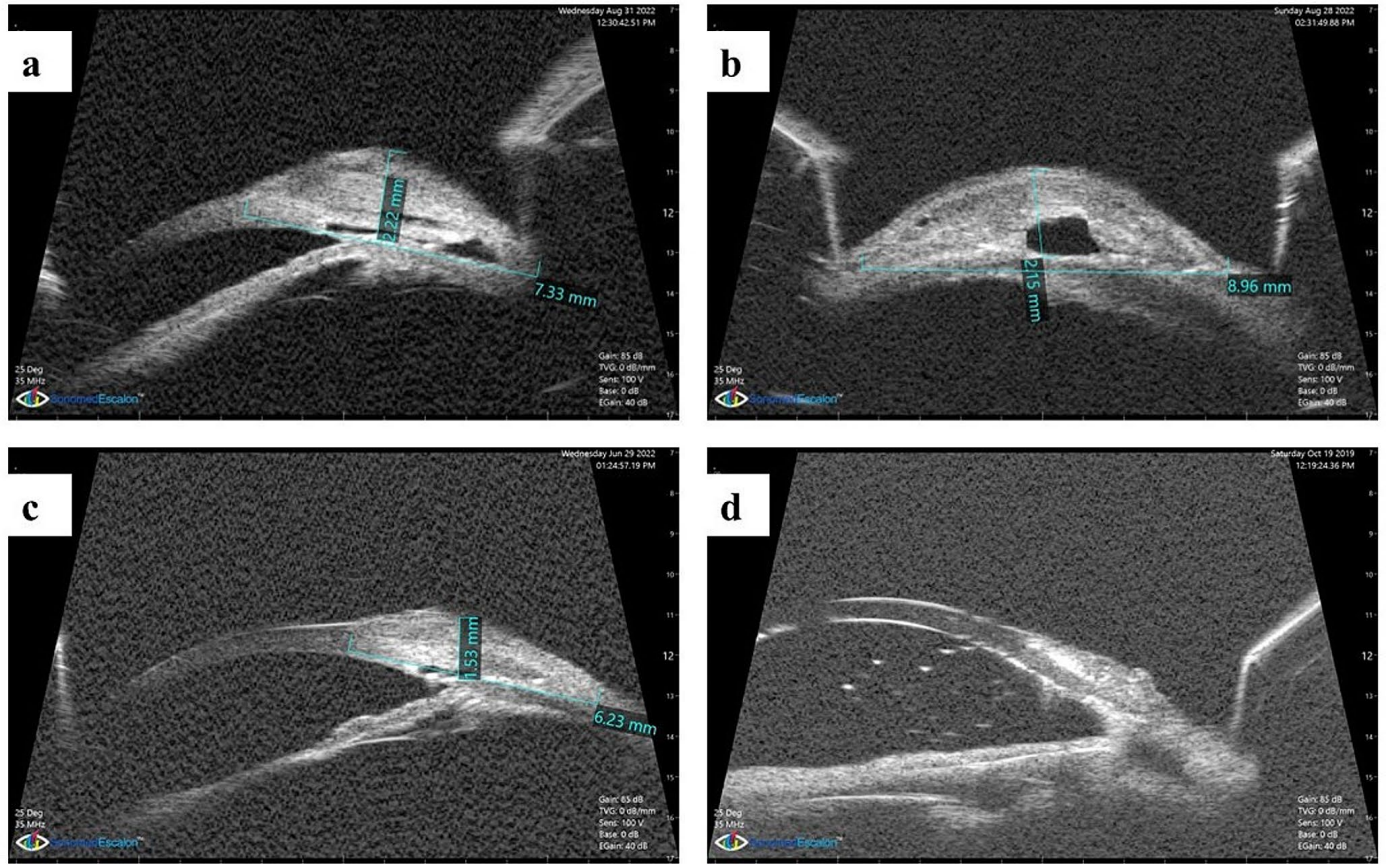
**a**, **b** UBM parameters for 2 cases classified as success. **c** UBM parameters of a case that needed needling. **d** UBM parameters of a case that was classified as failure

Mean post-operative IOP at 1 week, 1 month and 6 month follow ups was statistically significantly lower in group A (7.9 ± 1.3, 11.7 ± 2.2, and 13.3 ± 1.9 mmHg) than in group B (10.1 ± 4.6 and 14.1 ± 5.2 and 16.8 ± 4.1 mmHg) (*p* = 0.025, *p* = 0.041 and *p* = 0.001 respectively). However, the difference was not statistically significant at the 3 months post-op visit (*p* = 0.193) (Mean IOP of group A = 13.2 ± 3.8 mmHg and group B = 14.9 ± 4.2 mmHg). Also, on comparing the mean percentage of reduction of IOP compared to pre-operative IOP between the two groups, it was also larger in group A than in group B at the follow up post-operative visits (Fig. 3). The difference was statistically significant at the 6 month follow up (*p* = 0.027) where the percentage of reduction of IOP compared to pre-operative IOP at the 6 month follow up visit was 52.5% ± 14.3 in group A compared to 42.7% ± 14.2 in group B. (Table 2) (Fig. 4).

The drop and the percentage drop in the use of anti-glaucoma medications compared to the pre-operative antiglaucoma medications used was statistically significantly higher in group A (reduction of 2.7 ± 1.0 meds and % reduction of 86% ± 18) than in group B (reduction of 2.0 ± 1.1 meds and % reduction of 71% ± 35) (*p* value = 0.02 and 0.05 respectively) (Table 2).

**Table 2:** Comparative analysis between the 2 groups at different times throughout the study period:

	Group A				Group B				*p* value
	Mean	SD	Min	Max	Mean	SD	Min	Max	
Pre-op IOP in mmHg	30.3	8.2	22	46	30.1	4.4	21	40	0.786
IOP at 1 week in mmHg	7.9	1.3	5	10	10.1	4.6	3	25	***0.029***
IOP at 1 month in mmHg	11.7	2.2	8	18	14.1	5.2	7	27	***0.041***
IOP at 3 months in mmHg	13.2	3.8	8	21	14.9	4.2	11	27	0.193
IOP at 6 months in mmHg	13.3	1.9	9	16	16.8	4.1	11	27	**0.001**
% Drop of IOP after 1 week	71.9	8.7	56	89	67.6	13.2	38	90	0.111
% Drop of IOP after 1 month	58.7	13.4	25	83	53.2	15.6	11	69	0.140
% Drop of IOP after 3 months	53.9	15.5	17	83	49.9	12.0	27	66	0.389
% Drop of IOP after 6 months	52.5	14.3	30	76	42.7	14.2	14	66	***0.027***
6 months post-op meds	0.5	0.7	0	2	0.8	0.9	0	2	0.137
Drop in meds used in 6 months	2.7	1.0	1	4	2.0	1.1	0	4	***0.022***
% Drop in meds used in 6 months	86	18	50	100	71	35	0	100	***0.049***

Comparing the post-operative UBM parameters between the two groups, all the UBM parameters (The whole bleb length and height and the intrascleral lake length and height) were statistically significantly larger in group A than in group B at both 1 and 6 months (Table 3). Figure. 4 shows samples of cases of both groups that were classified as success, needed intervention or were classified as failure.

**Table 3:** Comparative analysis between the 2 groups regarding the UBM parameters

	Group A				Group B				*p* value
	Mean	SD	Min	Max	Mean	SD	Min	Max	
Bleb length at 1 month in mm	7.30	4.7	5.01	10.59	5.92	1.98	2.64	10.81	**0.008**
Bleb height at 1 month in mm	2.08	0.48	1.25	3.61	1.32	0.75	0.31	2.71	**< 0.001**
Intrascleral lake length at 1 month in mm	2.24	0.86	0.98	3.81	1.27	0.97	0	3.73	**0.001**
Intrascleral lake height at 1 month in mm	0.60	0.26	0.20	1.10	0.26	0.19	0	0.78	**< 0.001**
Bleb length at 6 months in mm	6.95	1.38	5.87	10.66	5.33	1.65	2.79	8.39	**< 0.001**
Bleb height at 6 months in mm	1.92	0.55	1.29	3.12	1.12	0.73	0.19	2.64	**< 0.001**
Intrascleral lake length at 6 months in mm	1.78	0.70	0	2.91	0.97	0.77	0	2.69	**0.001**
Intrascleral lake height at 6 months in mm	0.46	0.26	0	1.08	0.22	0.24	0	1.04	**0.004**

No statistically significant difference was found regarding the need for additional procedures like goniopuncture (4/20 in group A and 5/20 in group B) and needling (1/20 in group A and 2/20 in group B). Regarding success rates, no statistically significant difference was noticed between the 2 groups. In group A, 13/20 eyes were classified as complete success (65%), while 20/20 were classified as qualified success (100%), with no failures. While in group B, 11/20 eyes were classified as completer success (55%), while 19/20 were classified as qualified success (95%). And only 1/20 was considered a failure (5%).

**Fig. 5:**
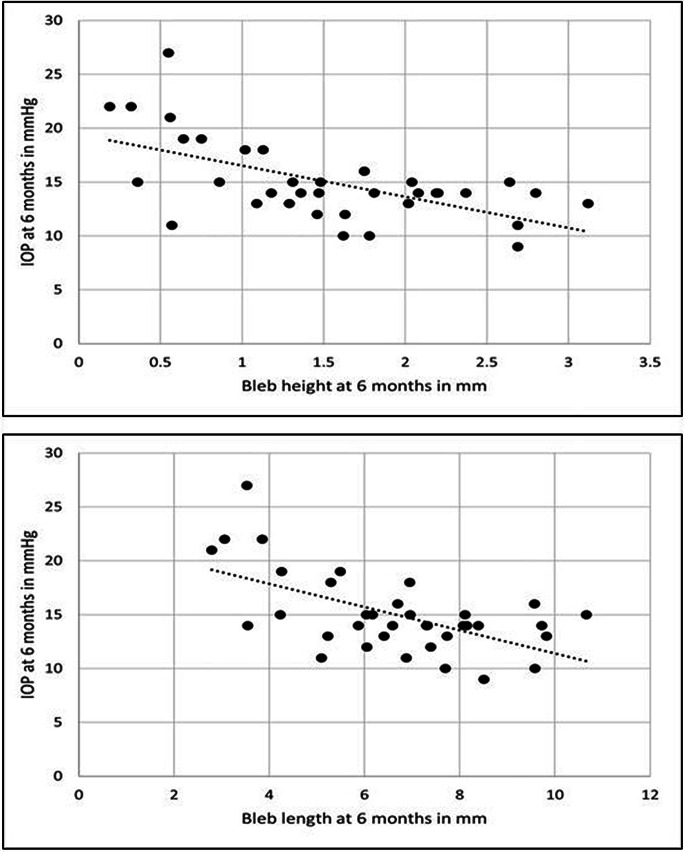
Correlation between bleb length and height with the IOP at 6 months Postoperatively

**Fig. 6:**
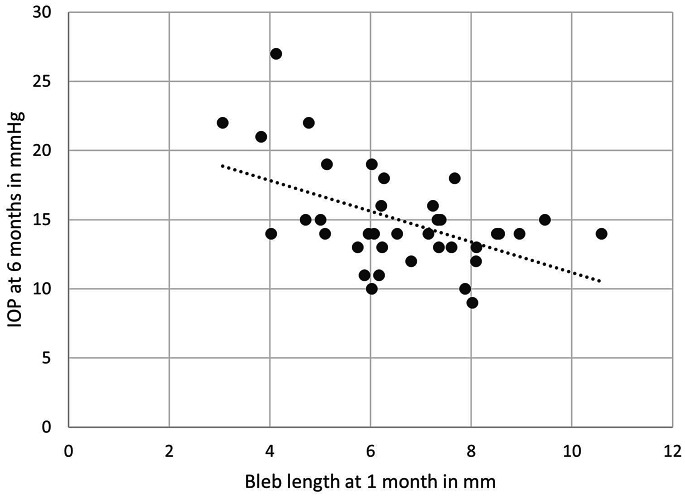
Correlation between bleb length at 1 month and IOP at 6 months postoperatively

When the relationship between IOP and UBM parameters was studied, we found strong statistically significant negative correlations between the IOP measurements at 1 month and the UBM parameters at 1 month. The strongest correlation was with the whole bleb anteroposterior length (*r*= -0.521, *p* = 0.001). Also at 6 months, strong statistically significant negative correlations were found between the IOP measurements at 6 months and all the UBM parameters at 6 months. The strongest correlations were with the whole bleb anteroposterior length and height (*r*= -0.585 and *r*= -0.597, *p* = < 0.001 for both respectively). Also, we found that the weakest correlation was with intrascleral lake height at both 1 and 6 month follow ups (*p* = 0.037 and *p* = 0.033 respectively) (Fig. 5). A strong statistically significant correlations were found between IOP at 6 months and all the UBM parameters at 1 month apart from intrascleral lake height. Again, the strongest correlations were with the whole bleb anteroposterior length and height (*r*= -0.500 and *r*= -0.523, *p* = 0.001 for both respectively) (Fig. 6) (Table 4).

**Table 4:** Correlation between IOP at 6 months follow up visit and different UBM parameters at 1 month and 6 months follow up visits

Correlation with 1-month UBM parameters	r	*p* value
Bleb length at 1 month	-0.500	**0.001**
Bleb height at 1 month	-0.523	**0.001**
Intrascleral lake length at 1 month	-0.357	***0.028***
Intrascleral lake height at 1 month	-0.281	0.088
Correlations with 6-months UBM parameters	*r*	*p* value
Bleb length at 6 months	-0.585	**< 0.001**
Bleb height at 6 months	-0.597	**< 0.001**
Intrascleral lake length at 6 months	-0.489	**0.002**
Intrascleral lake height at 6 months	-0.356	***0.033***

## Discussion

Ahmed’s sub-flap mattress suture improves the outcome of deep sclerectomy surgery through the following mechanisms: first, mechanical elevation of the limbal part of the superficial scleral flap, enhancing the percolation by minimizing the downward force on the TDM and allowing formation of the decompression space. Second, upward lifting of distal part of the superficial scleral flap, inducing some tissue gapping, thereby enhancing aqueous outflow to the subconjunctival space. Also, the proximal part of the suture lies just in front of the Schlemm’s canal allowing better identification of the TDM while performing YAG goniopuncture later if needed [8].

In our study, Ahmed’s sub-flap mattress suture group showed a statistically significantly lower post-operative IOP at 1 week, 1 month & 6 month (7.9 ± 1.3, 11.7 ± 2.2 and 13.3 ± 1.9 mmHg) than in conventional NPDS group (10.1 ± 4.6 and 14.1 ± 5.2 and 16.8 ± 4.1 mmHg) (*p* = 0.025, *p* = 0.041 and *p* = 0.001 respectively). However, the difference was not statistically significant at 3 months postoperatively (*p* = 0.193). Also, Adding Ahmed’s suture led to a statistically significant increase in the IOP reduction compared to pre-operative IOP at 6 months (52.5% vs. 42.7%, *p* = 0.027).

A previous study done by Abdelrahman et al., included 103 eyes (52 underwent DS with sub-flap mattress Ahmed’s suture versus 51 underwent standard DS). The Ahmed’s suture group had pre-op IOP of 27.4 ± 6.3 mmHg and 6-month post-op IOP of 12.6 ± 2.7 mmHg, drop of antiglaucoma meds use from 3.4 ± 1.0 pre-op meds to 0.2 ± 0.3 post-op meds, complete success rate of 81% and 100% qualified success rate. Goniopuncture was needed in 12% and needling in 4% of the cases. They showed that adding sub-flap mattress suture to deep sclerectomy increased the IOP lowering effect of deep sclerectomy from 40 to 52% of the pre-operative IOP at 6 months which agreed with our results where group A (with adding Ahmed’s sub-flap mattress suture) had nearly 53% reduction in IOP after 6 months compared to 43% in group B who underwent conventional deep sclerectomy [8].

Other modifications were done to enhance deep sclerectomy efficacy, many of them involved using sub-flap implants. Some of the implants are absorbable like: Ologen implants and others are non-absorbable like Esnoper clip [9, 10].

Elmekawy et al., studied the effect of adding Ologen implant to deep sclerectomy in a comparative study enrolling 40 eyes. In the Ologen implant group, pre-operative IOP was 19.3 mmHg and 6 months postoperative IOP was 12.8 mmHg with success rates 95% complete success and 100% qualified success at 6 months which are very comparable results to our study. It is important to note that preoperative IOP was much higher in our study (30.3 ± 8.2 mm Hg) indicating higher severity of the disease and still yielding same 6-month postoperative results which is in favor of Ahmed’s sub-flap mattress suture [9].

Loscos-Arenas et al. investigated adding Esnoper clip to NPDS in a study enrolling 27 eyes with pre-operative IOP of 26.6 ± 5.2 mmHg. Their 6 months postoperative IOP was 15.3 ± 5.2 mmHg, 25% of the patients needed goniopuncture, and the need for antiglaucoma drops dropped from 2.5 pre-operative meds to 0.3 postoperative meds. In our study, the pre-operative IOP was higher and the 6 months postoperative IOP was lower in Ahmed’s sub-flap mattress suture group. Also, goniopuncture was needed in 20% of cases, while post-op meds were slightly higher [10].

But to our knowledge, our study is the first to assess the surgical site in patients undergoing deep sclerectomy with Ahmed’s sub-flap mattress suture by UBM. We measured four UBM parameters: maximum antero-posterior whole bleb length & height, maximum intrascleral lake length and height at 1 and 6 months postoperatively. All the parameters were statistically significant larger in Ahmed’s sub-flap mattress suture group than in the conventional NPDS group.

We also found a strong negative correlation between the UBM parameters and IOP at 1 month and 6 months postoperatively. It was interesting to find a strong statistically significant correlations between IOP at 6 months and all the UBM parameters at 1 month except intrascleral lake height. The strongest correlations were with the whole bleb anteroposterior length and height which indicates that measuring the whole bleb anteroposterior length and height by UBM at 1 month post-operatively can be used a predictive indicator of IOP later at 6 months.

Perez et al., studied 47 eyes with previous NPDS done 5 years earlier with AS-OCT. They found significant negative correlation between IOP and intrascleral lake anteroposterior length & height which agrees with our study, but they didn’t find a significant correlation between IOP and transverse length of intrascleral lake or TDM thickness. We believe that AS-OCT can give higher resolution and more accurate measurements for the intrascleral lake but will not be able to measure the whole bleb dimensions [11].

Khairy et al. found no statistically significant correlations between IOP and any of the UBM parameters in a study that included 22 eyes with previous NPDS done around 12 months earlier. They used (intrascleral lake height & length and TDM thickness) [12].

Also, Cabrejas et al., studied 18 eyes undergoing deep sclerectomy and they found no significant correlations between IOP and the UBM measurements (Intrascleral lake anteroposterior and transverse lengths, intrascleral lake height) at 1, 3 and 6 months postoperatively. They found correlations between IOP and the presence of hyporeflective blebs, hypoechoic spaces in the supraciliary space and around the intrascleral lake, also Marchini et al., agreed on those three signs as a positive indicator of surgical success. The failure of these two studies to find correlations can be explained by the small sample size and that UBM is less accurate in assessing very small measurements of intrascleral lake [13, 14].

Fernandez-Buenaga et al., examined 60 eyes who underwent deep sclerectomy with 3 different implants (19 eyes with Sk-Gel implant, 22 eyes with Esnoper implant and 19 with Aquaflow implant) with UBM after mean of 15 months postoperatively. They found a statistically significant correlation between IOP and bleb height which agrees with our results [15].

Jankowska-Szmul et al., studied 40 eyes, 23 of them were successful CO2 laser-assisted sclerectomy surgery (CLASS) and 17 were considered as failures (IOP > 18mHg or needed re-operation) and examined them with AS-OCT. They found that the presence of scleral lake, intrascleral fluid was statistically significantly higher in eyes with successful surgeries and that scleral lake anteroposterior length and height measurements were becoming statistically significantly smaller with longer post-operative time over 1 year, but they couldn’t find a significant correlation between IOP and scleral lake height or anteroposterior length [16].

Mavrakanas et al., studied a series of 25 eyes with flat blebs after having NPDS with AS-OCT at a mean of 8 months postoperativelyy. They found statistically significant negative correlation between IOP and intrascleral bleb height which agrees with our results [17].

Aptel et al., studied 15 eyes in a prospective study performing deep sclerectomy with Ologen implant and followed them up for 3 months with both UBM and AS-OCT. They found that lower IOP correlated with bleb height and low TDM thickness based on UBM examination and lower IOP also correlated with thin bleb wall, large subconjunctival fluid spaces, and low bleb tissue reflectivity based on AS-OCT examination. This study recruited a small number of eyes but still agreed with our results regarding the bleb height [18].

Elmekawey et al., conducted a study recruiting 40 eyes (20 eyes underwent DS with Ologen implant and the other 20 eyes underwent conventional DS) and they were followed up for 6 months by UBM. They found a negative correlation between IOP and both the height and the extent of the bleb, which agrees with our results [9].

Our study was the only one that studied both the conjunctival bleb and the intrascleral lake parameters with UBM. And we found that using UBM can help to assess the surgical site of deep sclerectomy and predict the outcome, especially in larger measurements like whole bleb height and length. Our study also showed that adding the economical Ahmed’s sub-flap mattress suture was statistically significantly advantageous over the conventional DS regarding the IOP control and the UBM parameters.

### Drawbacks

Although, UBM is excellent in assessing the whole bleb dimensions, but adding AS-OCT can be useful to accurately measure the tiny intrascleral bleb dimensions. So, in future studies, we recommend combining both UBM for the whole bleb dimensions and AS-OCT for the small intrascleral bleb dimensions to assess deep sclerectomy surgical site.

## Data Availability

All the data included in the manuscript are available and would be revealed when issued.

## Code availability

Not applicable to our study.

## References

[1] Weinreb RN, Khaw PT (2004) Primary open-angle glaucoma. Lancet 363:1711–1720. 10.1016/S0140-6736(04)16257-0 15158634 10.1016/S0140-6736(04)16257-0

[2] King AJ, Hudson J, Fernie G, Kernohan A, Azuara-Blanco A et al (2021) Primary trabeculectomy for advanced glaucoma: pragmatic multicentre randomised controlled trial (TAGS). bmj 373:n1014. 10.1136/bmj.n1014 10.1136/bmj.n1014 PMC8114777 33980505

[3] Cairns JE (1968) Trabeculectomy: preliminary report of a new method. Am J Ophthalmol 66:673–679. 10.1016/0002-9394(68)91288-9 4891876

[4] Eldaly MA, Bunce C, ElSheikha OZ, Wormald R (2014) Non-penetrating filtration surgery versus trabeculectomy for open‐angle glaucoma. Cochrane Database Syst Reviews 2. 10.1002/14651858.CD007059.pub2 10.1002/14651858.CD007059.pub2 PMC11329888 24532137

[5] Rulli E, Biagioli E, Riva I, Gambirasio G, De Simone I, Floriani I, Quaranta L (2013) Efficacy and safety of trabeculectomy vs nonpenetrating surgical procedures: a systematic review and meta-analysis. JAMA Ophthalmol 131:1573–1582. 10.1001/jamaophthalmol.2013.5059 24158640 10.1001/jamaophthalmol.2013.5059

[6] Al Obeidan SA (2009) Nonpenetrating deep sclerectomy. Expert Rev Ophthalmol 4:299–315. 10.1586/eop.09.21

[7] Abdelrahman AM, Habib MM (2020) Sub-flap Mattress suture with Deep Sclerectomy: a novel step. J Glaucoma 29:127–129. 10.1097/ijg.0000000000001639 32826768 10.1097/IJG.0000000000001639

[8] Abdelrahman AM, Hassan LM, Habib MM (2023) Non-penetrating deep sclerectomy with the sub flap (Ahmed’s) suture: a 12-month comparative study. Eye 37:1308–1313. 10.1038/s41433-022-02102-6 35641822 10.1038/s41433-022-02102-6 PMC10169764

[9] Elmekawey H, Abdelrahman AM, Kotb MS, Mostafa DA (2020) Effect of filtering bleb dimensions on postoperative intraocular pressure in deep sclerectomy with collagen implant: a comparative study. Int Ophthalmol 40:7–12. 10.1007/s10792-019-01145-1 31321597 10.1007/s10792-019-01145-1

[10] Loscos-Arenas J, Parera-Arranz A, Romera-Romera P, Castellvi-Manent J, Sabala-Llopart A, De La Cámara-Hermoso J (2015) Deep sclerectomy with a new nonabsorbable uveoscleral implant (Esnoper-Clip): 1-year outcomes. J Glaucoma 24:421. 10.1097/ijg.0000000000000253 25836660 10.1097/IJG.0000000000000253 PMC4521906

[11] Pérez-Rico C, Gutiérrez-Ortíz C, Moreno-Salgueiro A, González-Mesa A, Teus M (2014) Visante anterior segment optical coherence tomography analysis of morphologic changes after deep sclerectomy with intraoperative mitomycin-C and no implant use. J Glaucoma 23:86–90. 10.1097/ijg.0b013e31829ea2c8 10.1097/IJG.0b013e31829ea2c8 24370813

[12] Khairy H, Atta H, Green F, Van der Hoek J, Azuara-Blanco A (2005) Ultrasound biomicroscopy in deep sclerectomy. Eye 19:555–560. 10.1038/sj.eye.6701558 15761488 10.1038/sj.eye.6701558

[13] Cabrejas L, Rebolleda G, Muñoz-Negrete F, Losada D (2011) An ultrasound biomicroscopy study of filtering blebs after deep sclerectomy with a new acrylic implant. Eur J Ophthalmol 21:391–399. 10.5301/ejo.2010.5843 21038309 10.5301/EJO.2010.5843

[14] Marchini G, Marraffa M, Brunelli C, Morbio R, Bonomi L (2001) Ultrasound biomicroscopy and intraocular-pressure-lowering mechanisms of deep sclerectomy with reticulated hyaluronic acid implant. J Cataract Refractive Surg 27:507–517. 10.1016/s0886-3350(00)00857-9 10.1016/s0886-3350(00)00857-9 11311615

[15] Fernández-Buenaga R, Rebolleda G, Casas-Llera P, Muñoz-Negrete FJ, Pérez-López M (2012) A comparison of intrascleral bleb height by anterior segment OCT using three different implants in deep sclerectomy. Eye 26:552–556. 10.1038/eye.2011.358 22241011 10.1038/eye.2011.358 PMC3325577

[16] Jankowska-Szmul J, Wylegala E (2018) The CLASS surgical site characteristics in a clinical grading scale and anterior segment optical coherence tomography: a one-year follow-up. J Healthc Eng. 10.1155/2018/5909827 29861883 10.1155/2018/5909827 PMC5976922

[17] Mavrakanas N, Mendrinos E, Shaarawy T (2010) Postoperative IOP is related to intrascleral bleb height in eyes with clinically flat blebs following deep sclerectomy with collagen implant and mitomycin. Br J Ophthalmol 94:410–413. 10.1136/bjo.2008.150318 20424212 10.1136/bjo.2008.150318

[18] Aptel F, Dumas S, Denis P (2009) Ultrasound biomicroscopy and optical coherence tomography imaging of filtering blebs after deep sclerectomy with new collagen implant. Eur J Ophthalmol 19:223–230. 10.1177/112067210901900208 19253238 10.1177/112067210901900208

